# Chemosynthesis enhances net primary production and nutrient cycling in a hypersaline microbial mat

**DOI:** 10.1093/ismejo/wraf117

**Published:** 2025-06-09

**Authors:** Francesco Ricci, Pok Man Leung, Tess Hutchinson, Thanh Nguyen-Dinh, Alexander H Frank, Ashleigh van Smeerdijk Hood, Vinícius W Salazar, Vera Eate, Wei Wen Wong, Perran L M Cook, Chris Greening, Harry McClelland

**Affiliations:** Department of Microbiology, Biomedicine Discovery Institute, Monash University, Clayton, VIC 3800, Australia; Securing Antarctica’s Environmental Future, Monash University, Clayton, VIC 3800, Australia; School of Geography, Earth and Atmospheric Sciences, University of Melbourne, Parkville, VIC 3010, Australia; Department of Microbiology, Biomedicine Discovery Institute, Monash University, Clayton, VIC 3800, Australia; Securing Antarctica’s Environmental Future, Monash University, Clayton, VIC 3800, Australia; Department of Microbiology, Biomedicine Discovery Institute, Monash University, Clayton, VIC 3800, Australia; Securing Antarctica’s Environmental Future, Monash University, Clayton, VIC 3800, Australia; Department of Microbiology, Biomedicine Discovery Institute, Monash University, Clayton, VIC 3800, Australia; Securing Antarctica’s Environmental Future, Monash University, Clayton, VIC 3800, Australia; Bayreuth Center of Stable Isotope Research in Ecology and Biogeochemistry (BayCenSI), University of Bayreuth, Bayreuth, Bavaria 95447, Germany; School of Geography, Earth and Atmospheric Sciences, University of Melbourne, Parkville, VIC 3010, Australia; Melbourne Bioinformatics, University of Melbourne, Parkville, VIC 3010, Australia; Water Studies Centre, School of Chemistry, Monash University, Clayton, VIC 3800, Australia; Water Studies Centre, School of Chemistry, Monash University, Clayton, VIC 3800, Australia; Water Studies Centre, School of Chemistry, Monash University, Clayton, VIC 3800, Australia; Department of Microbiology, Biomedicine Discovery Institute, Monash University, Clayton, VIC 3800, Australia; Securing Antarctica’s Environmental Future, Monash University, Clayton, VIC 3800, Australia; School of Geography, Earth and Atmospheric Sciences, University of Melbourne, Parkville, VIC 3010, Australia; Department of Structural and Molecular Biology, University College London, London WC1E6BT, United Kingdom

**Keywords:** chemosynthesis, microbialites, metabolic interactions, metagenomics, microbial mat, carbon fixation, isotopes, extreme environments

## Abstract

Photosynthetic microbial mats are macroscopic microbial ecosystems consisting of a wide array of functional groups and microenvironments arranged along variable redox gradients. Light energy ultimately drives primary production and a cascade of daisy-chained metabolisms. Heterotrophic members of these communities remineralise organic material, decreasing net primary production, and returning nutrients to the aqueous phase. However, reduced inorganic and one-carbon substrates such as trace gases and those released as metabolic byproducts in deeper anoxic regions of the mat, could theoretically also fuel carbon fixation, mitigating carbon loss from heterotrophy and enhancing net primary production. Here, we investigated the intricate metabolic synergies that sustain community nutrient webs in a biomineralising microbial mat from a hypersaline lake. We recovered 331 genomes spanning 40 bacterial and archaeal phyla that influence the biogeochemistry of these ecosystems. Phototrophy is a major metabolism found in 17% of the genomes, but over 50% encode enzymes to harness energy from inorganic substrates and 12% co-encode chemosynthetic carbon fixation pathways that use sulfide and hydrogen as electron donors. We experimentally demonstrated that the microbial community oxidises ferrous iron, ammonia, sulfide, and reduced trace gas substrates aerobically and anaerobically. Furthermore, carbon isotope assays revealed that diverse chemosynthetic pathways contribute significantly to carbon fixation and organic matter production alongside photosynthesis. Chemosynthesis in microbial mats results from a complex suite of spatially organised metabolic interactions and continuous nutrient cycling, which decouples carbon fixation from the diurnal cycle, and enhances the net primary production of these highly efficient ecosystems.

## Introduction

Microbial mat communities can form substantial carbonate structures termed microbialites, which represent the earliest macroscopic evidence of life on Earth [1, 2]. Microbial mats are also thought to have played a pivotal role in shaping Earth’s atmospheric composition [3, 4], not least their contribution to the oxygenation of the atmosphere following the evolution of oxygenic photosynthesis [3, 5], which amplified global biological productivity by 100–1000 fold [6]. Mat communities have furthermore been implicated as the source of large fluxes of reduced gases including carbon monoxide (CO), hydrogen (H_2_), and methane (CH_4_) into the atmosphere [6]. Though once ubiquitous in aqueous environments, today microbial mats are generally confined to extreme environments such as hypersaline lakes [7–11], where grazing metazoans and plants are typically absent [3]. The structure of microbial mat communities is strikingly conserved throughout geological history [5, 12], positioning modern mats as natural laboratories for studying the fundamental rules governing these ancient systems.

Microbial mats are hotspots of microbial diversity and biogeochemistry [13–15]. Light often plays a central role, driving organic carbon production, carbonate precipitation, and elemental cycling [5, 6, 11, 16]. Photosynthetic waste-products create new metabolic niches and fuel complex microbial interactions [3], including supporting the activity of aerobic heterotrophs [17–19]. Anaerobic microorganisms dominate internal microenvironments where oxygen has been depleted [6, 20, 21], including sulfate-reducing bacteria that use organic electron donors (e.g. acetate, sugars) to reduce sulfate to hydrogen sulfide (H_2_S) and diverse fermenters. Photosynthetic carbon fixation is often central to biomass production in these ecosystems [3, 22], but this is unlikely the sole primary production process. In the most reduced pockets, anaerobic autotrophs including methanogens and acetogens that grow via the Wood–Ljungdahl pathway (WLP) [23–25] can thrive; less well understood is the prevalence of chemosynthesis. Putative chemolithoautotrophs that encode genes for the oxidation of H_2_, CO, sulfide (S^2−^) and various inorganic compounds have been reported [26–28], but their ecological and biogeochemical roles within these ecosystems remain poorly understood. Although gene- and genome-resolved studies have provided preliminary insights into the functional potential of microbialite communities, direct activity-based evidence of the complex metabolic interactions that sustain these ecosystems remains limited.

Here we focus on the primary production and elementary cycling processes sustaining the biodiversity of calcifying microbial mats from a hypersaline lake. In photosynthetic mats, the classic view positions oxygenic photosynthesis as the leading process driving biological productivity [3, 6, 29, 30], yet the substantial amounts of reduced compounds such as H_2_, H_2_S, CO, and CH_4_ produced *in situ* [6, 21] could fuel diverse chemosynthetic processes. It is recognised that microbial mat communities can fix carbon through chemosynthetic pathways but a comprehensive understanding of how these processes are placed within the metabolic network of the community, and their relative contribution to primary production, is lacking. Using living microbialites from West Basin Lake, Victoria, Australia as a model system, we integrate gene- and genome-resolved metagenomics with spatial 16S rRNA gene amplicon sequencing, carbon isotope geochemistry, and phylogenetic analyses to quantify key metabolic activities and their contributions in supporting the community nutrient web.

## Materials and methods

### Characterisation of microbial mat organosedimentary structures

The eastern shoreline of West Basin Lake (38° 19′ 24.6468” S, 143° 26′ 51.8928″ E) hosts abundant microbialites and living microbial matgrounds [31]. Well-developed microbialite buildups and hardgrounds are exposed on subaerially-exposed benches up to several metres above the current lake level. Previous studies have documented extensive sub-aqueous, well-lithified living microbialites with considerable relief above the lake floor, especially in the adjacent East Basin Lake [31]. The living microbialites documented in this study occur in shallow water of around 0.5 m depth, and are best-developed in benches roughly 1–2 m from the current shoreline in West Basin Lake (Supplementary Fig.1). These living microbial matgrounds appear dark orange-red and have an irregular, undulating surface with a broad-scale relief of several centimetres. In cross sections, the mats have a dark orange-red film over a hard to friable, mineralised, cream-white uppermost layer of up to several millimetres in thickness (Supplementary Fig. 2). This mineralised layer has an irregular, pustular appearance and is composed of carbonate, likely hydromagnesite [31]. Underneath this, the mat consists of weakly lithified to unlithified mud with several thin colour zones over several millimetres depth (dark green, purple and grey), followed by up to several centimetres of light green mat (Supplementary Fig. 1; Supplementary Fig. 2). The sediment texture is commonly clotted and unlaminated. The substrate for the mat is dark, organic-rich mud that may have weak layering preserved through the presence of occasional coarse sediment laminae. Here we use the term ‘microbialite’ to define these microbial matgrounds, as they form mineralised structures with cm-scale relief above the lake floor. Although these modern and recent microbialites are not as extensively developed as the sub-aerially exposed microbialites, they do appear to form significant accretionary structures over time.

### Sample collection, processing, and physicochemical parameters

Growing microbialites were collected from West Basin Lake, a hypersaline (salinity 6.5–9.5%) inland crater lake located in Victoria, Australia. Microbialite samples were collected during three field trips in November 2022, March 2024, and May 2024 using pre-sterilised spades and transferred into pre-sterilised 5-gallon buckets containing lake water. After collection, samples were transported to the University of Melbourne or Monash University within 4 h. For biogeochemical and isotope analyses, samples were maintained in incubators at environmentally relevant temperatures under an 8 h:16 h light–dark cycle. Samples designated for DNA analysis were immediately stored at −80 °C. After 48 h, frozen samples were cleaved using a sterile hammer and chisel to isolate inner microbialite portions unaffected by field sampling. These procedures were implemented to minimise contamination during fieldwork and reduce disturbance to the microbial community. At first collection in November 2022, the water temperature (18.9 °C), dissolved oxygen (90%), pH (8.47) and redox potential (2.72 mV) were recorded. Irradiance at 30 cm (~650 μmol photons m^−2^ s^−1^) and 50 cm (~450 μmol photons m^−2^ s^−1^) below the water surface at noon was measured using a Walz Universal Light Meter (ULM-500) equipped with a Mini Quantum Sensor (LS-C). Physicochemical parameters for lakewater and microbialite depth-profile subsamples were measured (Supplementary Data 1) at the TrACEES Platform, University of Melbourne. Nitrate was measured at Water Studies, Monash University (Supplementary Data 1).

### 16S rRNA gene sequencing and community analysis

Total DNA was extracted from the layers of two different microbialite samples (three technical replicates per layer) using the DNeasy PowerSoil Kit (Qiagen, Hilden, Germany) on 0.5 g of material. Two sample-free negative controls were also included. Extracted DNA samples were sent to AGRF (Melbourne, Victoria) for library preparation, PCR-amplification and sequencing of the 16S rRNA gene V1-V3 regions on a MiSeq platform (Illumina), 2 × 300 bp paired-end reads. Sequences were processed using the QIIME2 pipeline v 2022.2 [32]. Primer sequences were trimmed using Cutadapt [33], whereas DADA2 was employed for merging forward and reverse reads, quality filtering, dereplication, and chimera removal [34]. Taxonomic classification was performed with QIIME2’s feature-classifier plugin. The SILVA v132 QIIME release was used for 16S rRNA gene taxonomy [35].

### Community DNA extraction and sequencing

Each of the three microbialite samples, comprising surficial and inner layers, was homogenised into a slurry. Total DNA was extracted from three different microbialite slurry samples (technical triplicate per sample for a total of nine samples) using the DNeasy PowerMax Soil Kit (Qiagen, Hilden, Germany) on 10 g of materials as per the manufacturer’s protocol. A sample-free negative control was also included. Extracted DNA samples were sent to AGRF (Melbourne, Victoria) for library preparation and sequencing on two lanes using a NovaSeq SP Flow-cell (Illumina), 2 × 150 bp for 500 cycles.

### Reads quality control, assembly, and binning

Across the three technical replicates of each microbialite sample, we obtained an average of over 32 million read pairs for sample 1 (range 935 779-92 420 867), over 113 million read pairs for sample 2 (range 100 612 691–129 406 257), and over 80 million read pairs for sample 3 (range 74 837 807–81 753 615). Reads quality control, assembly and binning were implemented within the Metaphor pipeline [36]. Specifically, raw reads derived from the nine metagenome libraries were quality-controlled by trimming primers and adapters, followed by artefacts and low-quality read filtering using fastp [37] with parameters *length_required 50*, *cut_mean_quality 30*, and *extra: —detect_adapter_for_pe*. The nine quality-controlled metagenomes were coassembled using MEGAHIT v1.2.9 [38] with default parameters. Given the similar nature and geographical provenience of the samples, coassembly was chosen to increase the recovery of rare microbial diversity and to create a less redundant set of contigs. Contigs shorter than 1000 bp were removed. Assembled contigs were binned using Vamb v4.1.3 [39], MetaBAT v2.12.1 [40], and CONCOCT v1.1.0 [41]. The three bin sets were then refined using DAS Tool v1.1.6 [42] and de-replicated using dRep v3.4.2 [43] with 95% ANI integrated with CheckM2 [44]. Bin completeness and contamination were estimated using CheckM2 [44]. After dereplication, we recovered 331 between medium (completeness >50%, contamination <10%) and high-quality (completeness >90%, contamination <5%) metagenome-assembled genomes (MAGs), according to MIMAG standards [45]. MAGs taxonomy was assigned according to Genome Taxonomy Database Release R214 [46] using GTDB-Tk v2.3.2 [47]. CoverM v0.6.1 [48] in genome mode was used to calculate the relative abundance of MAGs based on the metagenomic short reads.

### Metabolic annotation of metagenomic short reads and contigs

Paired-end reads from the samples collected in this study and public microbial mat sequences from Alchichica Lake [28], Socompa Lake [49], Highborne Cay [50], Shark Bay [51], and Rio Mesquites [52] recovered from the NCBI SRA under accession numbers PRJNA315555, PRJNA317551, PRJNA197372, PRJNA429237 and MG-RAST 4440067.3 respectively, were stripped of adapter and barcode sequences, then removed of contaminating PhiX and low-quality sequences (minimum quality score 20) using the BBDuk function of BBTools v.36.92 (https://sourceforge.net/projects/bbmap/). Resultant quality-filtered forward reads with lengths of at least 100 bp were searched for the presence of marker genes using the DIAMOND blastx algorithm [53]. Specifically, reads were compared against a custom-made reference databases [54] of 57 metabolic marker genes for energy conservation, carbon fixation, phototrophy, sulfur, nitrogen, andiron cycling, and H_2_, CO, CH_4_ cycling. A query coverage of 80% and an identity threshold of 80% for *psaA*, 75% for *hbsT*, 70% for *atpA, psbA, isoA, ygfK, aro*, 60% for *amoA, mmoA, coxL,* [FeFe]-hydrogenase*, nxrA, rbcL, nuoF*, and 50% for all others marker genes was used. The proportion of community members encoding each gene was calculated by normalizing the gene’s read count (measured in reads per kilobase million [RPKM]) against the average RPKM of 14 universal single-copy ribosomal marker genes. For annotation of binned and unbinned contigs, open reading frames (ORFs) predicted using Prodigal v2.6.3 [55] were annotated using DIAMOND blastp [56] homology-based searches against the above described database with the same thresholds.

### Phylogenetic analysis

Maximum-likelihood phylogenetic trees for archaeal and bacterial MAGs were built using GTDBtk [47] commands *identify* and *align* on MAGs. The archaeal and bacterial trees were built using IQ-TREE v2.3.6 [57, 58] with 1000 ultrafast bootstrap [59] using the LG + C10 + F + G and WAG+G20 models, respectively. MUSCLE [60] was used to align 36 AclB, 184 AcsB, and 140 RbcL amino acid sequences retrieved in the binned and unbinned contigs. AclB, AcsB, and RbcL maximum-likelihood phylogenetic trees were built using IQ-TREE v2.3.6 [57, 58] with 1,000 ultrafast bootstraps [59] and models LG + I + G4, LG + F + I + R6, and LG + R5 respectively. All trees were plotted using iTOL v6 [61] and edited in Illustrator v24.0.2.

### Chemical imaging

The O_2_ sensitive optode preparation included mixing 100 mg of polystyrene, 1.5 mg of indicator (PT (II) meso-tetra(pentafluorophenyl)porphine), 1.5 mg of reference (Macrolex yellow®) and dissolved in 1 g of solvent (Tetrahydrofuran) to form a cocktail. The O_2_ cocktail was knife-coated on dust-free polyester foil (goodfellow.com) and the final thickness of the coating was <2 μm. Once dry, the O_2_ sensitive optode was coated with an anti-refractory layer. The anti-refractory cocktail preparation included mixing 100 mg hydrogel D4, 100 mg carbon black and 1 g of 100% ethanol. The anti-refractory cocktail was knife-coated on top of the O_2_ sensitive optode and the final thickness of the coating was <3 μm.

The experimental setup included a modified digital single-lens reflex camera (Canon EOS 1000D) with its near-infrared (NIR) blocking filter removed and equipped with a Sigma 50 mm F2.8 EX DG Macro lens. An emission filter (Schott 530 nm, Uqgoptics.com) was fitted to the lens to detect oxygen fluorescence. Following the protocol described by Larsen et al. [62], O_2_ sensitive optode were excited by four high-power blue LEDs (l-peak = 445 nm, LXHL-LR3C, Luxeon, F = 340 mW at IF = 700 mA) combined with a 470 nm short-pass filter (blue dichroic colour filter, Uqgoptics.com). Microbialite sample cross-sections were illuminated using a Schott Leica KL 2500 LCD Cold Light Source. All components were synchronized via a trigger box (https://imaging.fish-n-chips.de) and controlled using the custom software Look@RGB. Each planar optode was calibrated individually in an aquarium maintained at a constant seawater temperature of 20 ± 1°C in a dark room. The calibration range for the O_2_ sensitive optode was 0–360 μmol L^−1^.

All experiments were conducted in a dark room at a constant 20 ± 1°C to resemble the lake temperature at the time of sampling. Three microbialite samples were cut using a diamond saw exposing their cross-sections. Each sample was placed in a 4 L glass aquarium pressing on the O_2_ sensitive optode, which was attached to the aquarium side. Following overnight acclimation in the aquaria, microbialite sample cross-sections were illuminated with ~450 μmol m^−2^ s^−1^ of light from above mimicking *in situ* sunlight. Irradiance levels in the experimental setup for defined lamp settings were measured using a Walz Universal Light Meter (ULM-500) equipped with a Mini Quantum Sensor (LS-C). Image sequences capturing O_2_ dynamics across the microbialite cross-sections were taken every 5 min. Lake water in the aquarium was aerated with an aeration stone connected to an air pump.

Data analysis was conducted using ImageJ v1.53K. Each image was separated into Red, Green, Green2, and Blue RAW TIFF channels. The ImageJ plugin Ratio Plus was used to calculate the ratio of the Red to Green channels (R/G). The resulting ratio images were colour-coded using the “Fire” lookup table to visualize O_2_ dynamics. Calibration was performed using the Curve Fitting function, applying an exponential fit with offset for O_2_​, based on planar optode calibration values. Brightness and contrast settings were adjusted to display minimum and maximum values of 0–360 μmol L^−1^ for O_2_​ images. To reduce the effect of water seeping between the O_2_​ optode and the microbialite cross-section, and to accurately quantify microbial O_2_​ production, we subtracted the first image of each experiment from all subsequent images using the Image Calculator function. To identify the photosynthetic regions on the microbialite sample cross-sections, we overlaid images with the highest O_2_ production onto microbialite cross-section images. The portions of each microbialite sample that showed O_2_ production were identified as regions of interest (ROI). Subsequently values were extracted from the ROI. The control sample was an image sequence of ROI measuring O_2_ dynamics in a deeper, anoxic microbialite cross-section portion. Net photosynthesis (PN) and apparent dark respiration (RD) were estimated by subtracting images taken with a 5 minutes interval when O_2_ production and respiration were the highest, respectively. Gross photosynthesis was estimated as PG = PN + |RD|.

### Ex situ biogeochemical measurements

We conducted incubation experiments to evaluate the aerobic and anaerobic metabolism of microbialite communities. Each experiment was performed in triplicate, with three independent microbialite samples per replicate. Control samples were prepared by gamma radiation followed by one autoclave cycle at 121°C for 30 minutes. These controls confirmed that the observed element dynamics were attributable to biotic processes.

Aerobic and anaerobic incubations, including trace gases, S^2−^ and Fe^2+^ additions, were set up in 120 ml serum vials containing 50 ml of 0.22 μm-filtered lake water and ~10 g of microbialite slurries. The vials were sealed with butyl rubber septa. For aerobic incubations, the headspace was left with ambient air, whereas anaerobic incubations were flushed with helium for 10 minutes to remove O_2_. Nitrate (1.5 mM NO_3_^−^) was then added as an electron acceptor to all anaerobic incubations except those targeting S^2−^ production, which relied on the endogenous sulfate present in the samples. The nitrate concentration was selected to ensure electron acceptor availability and may exceed typical *in situ* levels, potentially leading to elevated activity rates. The weights of microbialite used in each incubation were recorded and used to normalise calculations.

Trace gas incubations were supplemented with 10 ppm H_2_, CH_4_, and CO in the headspace. Sampling of the headspace began immediately after the addition of electron donors and acceptors, with 2 ml of gas extracted at variable time intervals. In anaerobic vials, the sampled gas volume was replaced with He. Gas concentrations were analysed by gas chromatography using a pulsed discharge helium ionization detector (model TGA-6791-W-4 U-2, Valco Instruments Company Inc.), with calibration based on certified standard mixtures of H_2_, CH_4_, and CO (0, 10, 100 ppm in N_2_, BOC Australia).

In anaerobic S^2−^ and Fe^2+^ incubations, either 100 μM Na₂S·9H₂O or 6 mM FeCl₂ was added to helium-purged 120 ml serum vials. At each timepoint, 3 ml of water was sampled and filtered through 0.45 μm pore-size filters. For S^2−^ analysis, 2 ml of the filtered sample was preserved with zinc acetate, whereas for Fe^2+^ analysis, 1 ml was preserved with ferrozine. Both S^2−^ and Fe^2+^ concentrations were measured using a GBC UV–Visible 918 spectrophotometer, following established methods [63].

Incubations for nitrification were prepared in uncapped 250 ml Schott bottles containing 100 ml of 0.22 μm-filtered lake water, 100 μM NH_4_^+^, and ~10 g of microbialite sample. At each sampling timepoint, 10 ml of water was filtered through 0.45 μm pore-size filters and stored frozen until further analysis. The filtered samples were analysed for NO_x_ (NO_2_^−^ + NO_3_^−^) concentrations using a Lachat QuikChem 8000 Flow Injection Analyzer (FIA) in accordance with APHA methods [64]. For oxygen consumption measurements, incubations were prepared in 120 ml vials containing 100 ml of 0.22 μm-filtered lake water and ~10 g of microbialite slurry and kept in darkness. Dissolved O_2_ concentrations were monitored using a FireSting oxygen probe (PyroScience) until the incubations approached anoxic conditions.

### ^14^C incorporation analysis

0.25 g of homogenized microbialite sample with 1 ml of 0.22 μm-filtered lake water were prepared in 7 ml scintillation vials with ambient air headspaces. Radiolabeled sodium bicarbonate solution (NaH_14_CO_3_, Perkin Elmer, 53.1 mCi nmol^−1^) was added to an approximate concentration of 0.1 μM. Triplicates of each sample were prepared and subjected to five different conditions, namely light (40 μmol m^−2^ s^−1^), dark, dark + H_2_ (100 ppm), dark + S^2−^ (800 μM), and dark + NH_4_^+^ (1 mM), and incubated for 5 days. These experiments aimed at capturing both aerobic and anaerobic carbon incorporation metabolisms. According to parallel O_2_ measurements, incubation experiments transitioned to anoxic after ~24 hours. After the incubation period, concentrated HCl was added dropwise to each vial and left for 24 hours with intermittent shaking to ensure excess unbound dissolved inorganic carbon (DIC) was acidified and released as ^14^CO_2_. HCl was added equally to all vials until bubble production ceased before they were placed at 60°C under a heat lamp to dry. When dry, 7 ml of scintillation liquid (EcoLume™, MP Biomedical) was added and ^14^C measured on an automated liquid scintillation counter (Tri-Carb 2810 TR, Perkin Elmer). Photosynthetic ^14^C incorporation values were adjusted to account for the photosynthetic ROI areas present on the 0.25 g portion of microbialite used in the light treatment. Assuming that the photosynthetic ROI areas identified through chemical imaging represent the microbialite sample regions performing oxygenic photosynthesis, we normalised by applying the ratio of photosynthetic ROI area of microbialite sample 3 which had the largest photosynthetic surface area (photosynthetic ROI sample 1: 3.11%, sample 2: 2.73%, and sample 3: 6.35%) to total microbialite area. We developed a hierarchical Bayesian model to analye ^14^C incorporation rates under different conditions. The model is implemented in Stan and R. The model consists of N total observations, with I samples, J conditions, and K replicates for each sample condition combination. As the dark + H_2_ condition experiment failed for microbialite C, we ran two models: one excluding microbialite C and included all conditions (where I = 2, J = 5, K = 3, and N = 30), and one excluding the dark + H_2_ condition and including all samples (where I = 3, J = 4, K = 3, and N = 36), so that in each case the model can be fitted to a complete dataset. The likelihood function is given by:


$$ {y}_{ijk}\sim N\left({\alpha}_i+{\beta}_j,{\sigma}^2\right), $$

where ${\alpha}_i$ describes the sample specific effect for the i^th^ sample, ${\beta}_j$ describes the condition specific effect for the j^th^ sample, and $\sigma^{2}$ is the unexplained variance in the data. The Dark condition (j = 1) was prescribed to be the control by setting ${\beta}_1=0.$Weakly informative priors were used. Priors for ${\alpha}_i$ ($i\in \left\{1:I\right\}$) and ${\beta}_j$ ($j\in \left\{2:J\right\}$) were specified as normal distributions with variances an order of magnitude larger than the variance of the total dataset. The prior for $\sigma$ was a Cauchy distribution. $\beta$ represents posterior probability distributions with credible intervals defined between the 2.5^th^ and 97.5^th^, and the 25^th^ and 75^th^ percentiles.

### Natural carbon stable isotope measurements

Naturally occurring stable isotopic compositions were determined to provide insight into possible pathways contributing to the formation of organic matter. For each sample, 12 replicates were prepared by chiselling 12 different regions of the microbialites. Approximately 1.5 g of the chiseled materials were dried at 70°C for 2 days, powder-homogenized with a clean mortar and pestle, and further dried at 70°C for 2 days, before shipping to the Bayreuth Center for Stable Isotope Research in Ecology and Biogeochemistry (BayCenSI), University of Bayreuth, Germany for isotopic analyses.

Stable isotope ratios of carbon in the sample are expressed in δ-notation:



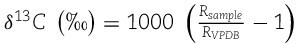



where *R_sample_* is the isotope ratio of ^13^C to ^12^C in the sample and *R_VPDB_* is the ratio in the Vienna Pee Dee Belemnite standard [65, 66].

The relative isotopic composition of the total carbon content in the microbialites was measured using Elemental Analysis-Isotope Ratio Mass Spectrometry (EA-IRMS). To isolate the organic fraction, samples were acidified with one droplet of 85% orthophosphoric acid (Analytical Reagent Grade, Fisher Scientific GmBH, Schwerte, Germany) in silver capsules (5 × 9 mm, IVA- Analysentechnik GmbH & Co. KG, Meerbusch, Germany). The reaction was allowed to proceed at room temperature for at least 48 hours. After this period, the capsules were carefully folded and packed into tin capsules (5 × 12 mm, IVA- Analysentechnik GmbH & Co. KG, Meerbusch, Germany).

The samples were introduced into the oxidation oven of the EA using a helium-purged autosampler. O_2_ gas was simultaneously injected to the carrier gas (helium at a flow rate of 100 ml min^−1^) to facilitate oxidation in a Fisons-EA-1108 CHNS-O Element Analyzer equipped with a dual reactor setup at 1020 °C for oxidation and 650 °C for reduction. The GC column (Porapak Q, 80/100, 1.8 m, 2 mm ID) was kept isothermally at 90°C, allowing to isolate the chromatographic peak of CO_2_ from accompanying combustion products. The EA was interfaced through an open split (ConFlo IV universal interface, Thermo Scientific, Bremen, Germany) to an IRMS (Delta V Advantage, Thermo Fisher Scientific). The isotopic ratio of the CO_2_ peak was determined through integration and internal calibration against a known reference gas using Isodat 3.0 (Thermo Scientific, Bremen, Germany). After export of the chromatographic areas and isotope ratios; instrument drift, linearity, δ-scale, and the carbon content of the samples was monitored and corrected using a set of external international standards (USGS-62, USGS-63, IAEA-CH7, IAEA-610) spanning the range of measurements.

The fractionation factor (ε_DIC-organic_) between the dissolved inorganic carbon (DIC) and organic carbon of the microbialite samples was approximated using the formula:


\begin{equation*} \varepsilon_{\text{DIC-organic}}=\lambda^{13}\text{C}_{\text{DIC}} - \lambda^{13}{\text{C}}_{\text{organic}} \end{equation*}

where $\delta$^13^C_DIC_ was estimated using a factor of 2.7‰ reported for the carbon fractionation between aragonite and DIC [67]:


\begin{equation*} \lambda^{13}\text{C}_{\text{DIC}} - \lambda^{13}{\text{C}}_{\text{carbonate}}-2.7 \end{equation*}

### Statistics and visualization

Downstream statistical analyses were performed in RStudio (version 1.2.5033) using R packages decontam [68], ggplot2 [69], phyloseq [70], and vegan [71]. Illustrator v24.0.2 was used for figure editing.

## Results and discussion

### Taxonomically and metabolically diverse microbes control a complex nutrient web in living microbialites

**Figure 1:**
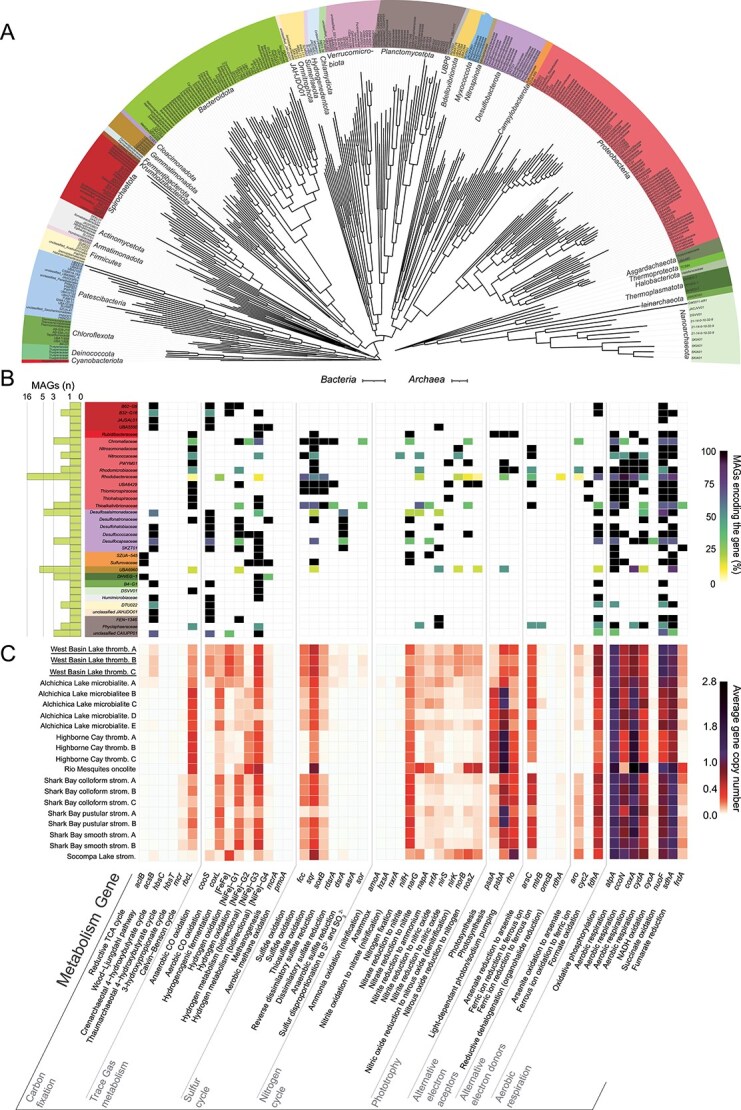
(**A**) Maximum-likelihood genome tree depicting the taxonomic diversity of 331 archaeal and bacterial metagenome-assembled genomes (MAGs), built with 1,000 ultrafast bootstrap replicates using the LG + C10 + F + G and WAG+G20 models, respectively. (**B**) Metabolic potential of microbes co-encoding energy acquisition enzymes with carbon fixation pathways. The side bargraph shows the number of MAGs in each microbial family (GTDB taxonomy). (**C**) the bottom heatmap shows the abundance of each gene in the metagenomic short reads in West Basin Lake samples (underlined) and across 17 publicly available microbialite community metagenomes from five global sites encompassing Alchichica Lake, Socompa Lake, Highborne Cay, Shark Bay, and Rio Mesquites. Homology-based searches were used to identify signature genes encoding enzymes associated with metabolic pathways. To infer abundance, read counts were normalized to gene length and the abundance of single-copy marker genes. *Bacteria* and *Archaea* phylogenetic trees scale bars are both 0.1.

We deeply sequenced nine metagenomes across three microbialite communities from West Basin Lake, Victoria, Australia. This effort resulted in the recovery of 331 medium- to high-quality metagenome-assembled genomes (MAGs) dereplicated at the species level using a 95% ANI threshold. These MAGs depict a wide microbial diversity spanning 40 bacterial and archaeal phyla (Fig. 1). Many of these microbes represent elusive and rare lineages, such as *Cloacimonadota*, *Krumholzibacteriota*, *Hydrogenedentota*, *Omnitrophota*, *Sumerlaeota,* and candidate phyla *JAHJDO01* and *UBP6*. Representatives of these lineages have been previously found in extreme environments including an Antarctic lake [72], deep-sea trenches [73], geothermal springs [74], and hypersaline microbial mats [75, 76]. The most abundant phyla were *Proteobacteria* (81 MAGs) and *Bacteroidota* (56 MAGs; Fig. 1A). *Cyanobacteria* are often assumed to be key members of microbialite-hosted ecosystems, though we only retrieved one medium-quality MAG of *Halothece* (1.0 ± 0.3% of mapped reads), and 18 MAGs capable of anoxygenic chlorophototrophy (Fig. 1A). Millimeter-scale 16S rRNA gene community profiling detected 30 cyanobacterial sequences, which were present at considerable relative abundance in the analysed samples (12.9 ± 10.6%, Supplementary Data 2). *Archaea* are also significant members, represented by 18 MAGs spanning *Asgardarchaeota*, *Halobacteriota*, *Iainarchaeota*, *Nanoarchaeota*, *Thermoplasmatota*, and *Thermoproteota* (Fig. 1A). Several major members of diatoms and red and green algae were also identified in our samples (Supplementary Data 2), consistent with observations in other modern microbialite communities [77–79].

Screening of key metabolic pathways in the MAGs revealed extensive metabolic diversity within West Basin Lake microbialite communities (Fig. 1B). Most microbes appeared to be facultative anaerobes that use organic carbon as an electron donor, with 63% of genomes encoding at least one terminal oxidase for aerobic respiration and 49% encoding at least one reductase for anaerobic respiration (Supplementary Data 3). The widespread capacity to oxidize various inorganic compounds (53% of genomes), including H_2_, S^2−^, and CO, for supplemental energy likely allows organic carbon to be efficiently used for essential anabolic processes (Supplementary Data 3). Some MAGs include genes encoding enzymes for utilising multiple energy sources. For instance, a *Thiohalophilus* MAG, family *UBA6439*, encodes the Calvin Benson-Bassham (CBB) cycle with uptake [NiFe]-hydrogenases, sulfide:quinone oxidoreductase, thiosulfohydrolase, reverse dissimilatory sulfite reductase, and iron oxidising cytochrome (Fig. 1A, B).

At West Basin Lake, multiple lines of evidence suggest that most organic carbon is produced in situ. First, the lake is not connected to any inlet and lacks aquatic vegetation. Second, moderate concentrations of organic carbon were present in the water (4.83 ± 0.06 mg/L), whereas dissolved organic carbon within the microbialites increases from 3.09 ± 0.43 mg/L in shallow to 5.60 ± 0.36 mg/L in deeper layers (Supplementary Data 1). This vertical dissolved organic carbon gradient suggests localised production or accumulation within the microbialite structure rather than passive deposition. Third, 13.6% of the MAGs include genes associated with carbon fixation (Fig. 1A, B). Specifically, 19 MAGs encoded the Wood-Ljungdahl Pathway (WLP; pathway completeness 77 ± 14%; Supplementary Data 4), whereas 16 MAGs encode the CBB cycle (pathway completeness 67 ± 30%; Supplementary Data 4) and also harbour genes associated with the oxidation of various inorganic substrates (Fig. 1; Supplementary Data 3), which likely provide energy and reductants to support chemolithoautotrophy. The WLP was encoded by diverse microbial taxa such as the *Phycisphaeraceae* and *FEN-1346* (*Planctomycetota*), *Humimicrobiaceae* (*Actinomycetota*), and JAJSAL01 (*Spirochaetota*; Fig. 1A, B). Several MAGs including the purple sulfur bacteria *Chromatiaceae,* the purple non-sulfur bacteria *Rhodobacteraceae,* and the obligately alkaliphilic *Desulfonatronaceae* may utilize S^2−^ as an electron donor via sulfide:quinone oxidoreductase and to a lesser extent H_2_ via group 1 and 3 [NiFe]-hydrogenases to fix CO_2_ via the CBB (Fig. 1). In addition to oxygenic photosynthesis by *Cyanobacteria* and several microalgae of phyla *Cercozoa*, *Chlorophyta*, *Gyrista,* and *Rhodophyta* (Supplementary Data 2), two *Proteobacteria* and a high-quality *Gemmatimonadota* MAGs have the genomic capacity to harvest light (*via* photosystem II), oxidize S^2−^, and fix carbon (*via* the CBB; Supplementary Data 3; Supplementary Data 4). Lastly, we found six MAGs encoding the reductive tricarboxylic acid cycle (rTCA, pathway completeness 66 ± 13%; Supplementary Data 4), three of which belonged to the enigmatic group 3 [NiFe]-hydrogenase-encoding *Thermoplasmatota* class *E2* (Fig. 1A, B).

To place the inferred metabolic traits supporting energy conservation and primary production in the microbialite communities of West Basin Lake in context, we compared these metagenomes to those of five other global sites previously sequenced. Together, these samples span various mat structures (thrombolite, oncolite, and stromatolite) and wide geographical ranges (Mexico, Argentina, Bahamas, and Australia; Fig. 1C; Supplementary Data 5). The capacity for carbon fixation is consistently high across microbialite communities represented in these datasets. In West Basin Lake, the CBB (*rbcL* carried by 19.6 ± 2.1% of the community) and the WLP (*acsB* 9.9 ± 2.4%) were predominant. In the comparison datasets, the CBB was also the major pathway (32.5 ± 17.0%), whereas the WLP and 3-hydroxypropionate cycle showed occasional dominance in specific samples (Fig. 1C). The potential to oxidise inorganic substrates was consistently present across sites, possibly enabling continuous energy acquisition throughout the diel cycle. In addition to corroborating previous findings [26–28] that identify S^2−^ as a major energy source (*sqr*: West Basin Lake 60.9 ± 4.0%, global 40.1 ± 20.9%), we show that H_2_ oxidation is also a widespread metabolism (Group 1–3 [NiFe]-hydrogenase: West Basin Lake 24.5 ± 20.3%, global 22.2 ± 16.0%). The capacity for CO and arsenite oxidation–ancient metabolic traits–was also substantial across all microbial communities (*cooS* & *coxL*: West Basin Lake 17.7 ± 3.9%, global 11.2 ± 11.7%; *aro*: West Basin Lake 11.5 ± 3.2%, global 10.1 ± 7.3%). Conversely, the abundance of photosynthesis genes showed marked variations across samples. West Basin Lake had moderate capacity for chlorophototrophy (*psaA, psbA:* 21.9 ± 17.3%) and rhodopsin-mediated phototrophy (*rho:* 30.5 ± 7.0%), whereas the global comparison datasets exhibited broader ranges (*psaA, psbA:* 12.6–141.3%; *rho:* 13.1–113.5%). This variability emphasizes that microbialite phototrophic communities are affected by local environmental conditions [80], for instance, increased salt concentration is known to influence the fitness of eukaryotic and bacterial phototrophs in diverse aquatic environments [81, 82]. Overall, our findings demonstrate that the genetic potential for energy conservation and carbon fixation in West Basin Lake microbialites is broadly representative of other microbialite communities that have been studied globally. The high metabolic flexibility observed in these ecosystems may enable them to act as efficient engines of biological productivity, supporting diverse and dynamic microbial communities.

### Tight microbial interactions support intricate biogeochemical cycles

Consistent with previous work [14, 26, 27, 52, 80], our findings reveal that the microbialite communities of West Basin Lake are characterised by high metabolic diversity. Millimetre-scale community analysis revealed high microbial richness throughout the entire microbialite structure (Supplementary Fig.2), with each layer harbouring divergent microbial communities (PERMANOVA: *F* = 2.65, *P <* .001; Supplementary Data 2). Most microbial taxa were present in low abundance, with 18 out of 25 phyla having a relative abundance of less than 1% (Supplementary Data 2). In contrast, sequences associated with the *Proteobacteria* (59.5 ± 16.8%) were distributed across the whole microbialite structure but were most prevalent in the upper orange layer (Supplementary Data 2). *Planctomycetota* and *Desulfobacterota*, key nitrogen and sulfur cyclers respectively, were also present throughout the whole microbialite structure but at much lower relative abundance (Supplementary Data 2). Observations at finer taxonomic resolution revealed that sequences associated with sulfur-oxidising *Campylobacterales*, phototrophic *Cyanobacteriales,* and obligately anaerobic *Spirochaetales* were ubiquitous (Supplementary Data 2), whereas other taxa such as fermenters in the *Clostridiales* were confined to deeper anoxic niches (grey layer—Supplementary Data 2). The diverse physiological requirements of microbes in each layer suggest that West Basin Lake microbialites microenvironments physicochemistry is likely shaped by their unlaminated structure and may change over time, likely on a diel cycle. While at night anaerobic metabolisms possibly prevail, the expansion of oxic pockets during the day as a result of oxygenic photosynthesis may foster aerobic metabolisms, thus allowing diverse functional guilds to coexist throughout the microbialite structure.

**Figure 2:**
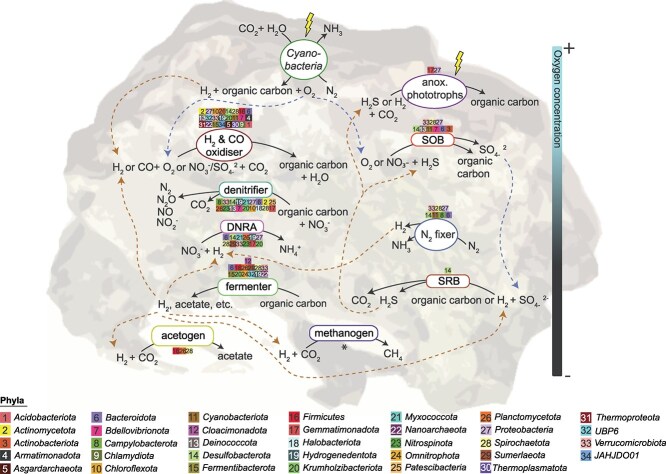
Conceptual overview of the community metabolic interactions based on genome- and gene-resolved data of the dominant microbial guilds within the microbialite communities of West Basin Lake. This overview represents the inferred metabolic pathways and interactions based on the genomic content of these guilds. Dashed lines indicate the direction of electron acceptors and donors. The graphic is an artistic generalisation of the data and should not be interpreted as an exact depiction of the microbial community structure. Asterisk (*) denotes that specific metabolic marker genes were exclusively recovered from metagenomic short-read data. Lightning bolts represent light energy. The background image shows a microbialite cross section.

We developed a conceptual overview of the community metabolic interactions to elucidate the array of molecular exchanges occurring within microbialite communities (Fig. 2). H_2_ emerges as a central molecule, consistent with previous studies of microbial mats and hydrothermal vent communities [83–85]. In microbialite communities, H_2_ is primarily produced through hydrogenogenic fermentation of photosynthetically- and chemosynthetically-derived organic carbon via diverse group 3 [NiFe]-hydrogenases and [FeFe]-hydrogenases, encoded by 90 and 55 MAGs respectively (Fig. 2). The genetic potential for other fermentation pathways is also high, with many MAGs carrying marker genes associated with fermentative production of acetate (*acdA*, *ack*, *pta*), formate (*pflD*), and lactate (*ldh*; Supplementary Data 6). Diazotrophic *Cyanobacteria* and members of six other phyla (16 MAGs) may also contribute to H_2_ release as an obligate by-product of the nitrogenase reaction. Upon diffusion into aerobic environments, H_2_ is readily used by the abundant gas oxidisers spanning 25 phyla (106 MAGs; Fig. 2), including for carbon fixation (Supplementary Data 3). Anaerobic processes also likely utilie much of the H_2_, including coupling H_2_ oxidation to denitrification (109 MAGs; Supplementary Data 3) and dissimilatory nitrate reduction to ammonium (55 MAGs; Supplementary Data 3). Hydrogenotrophic acetogens were found across each microbialite sample (2.6 ± 0.1% of mapped reads). H_2_ also fuels methanogenesis and sulfate reduction to varying degrees, contributing to the production of CH_4_ and S^2−^ (Fig. 2). However we could not reconstruct any methanogen MAG due to their low abundance in the community, as reflected by the low proportion of reads for the methanogenesis marker gene *mcrA* (0.12 ± 0.0% of the community), which in our dataset is predominantly affiliated with the genera *Methanohalophilus* and *Methanolobus* (Supplementary Data 7). Aerobic and anaerobic CO oxidation are significant processes within microbialite communities (34 MAGs; Fig. 2), supporting energy conservation and carbon fixation in eight phyla (Supplementary Data 3). A total of 78 MAGs, representing chemolithotrophic and photolithotrophic microorganisms across nine phyla, carry genes associated with S^2−^ oxidation under both aerobic and anaerobic conditions (Fig. 2; Supplementary Data 3). This capability likely provides a substantial ecological advantage, facilitating the persistence of these microorganisms in environments characterised by dynamic redox fluctuations and intense resource competition, as seen in other S^2−^ rich ecosystems such as coastal marine sediments [86] and hydrothermal vents [87].

### Microbialite communities mediate broad aerobic and anaerobic elemental cycling

**Figure 3:**
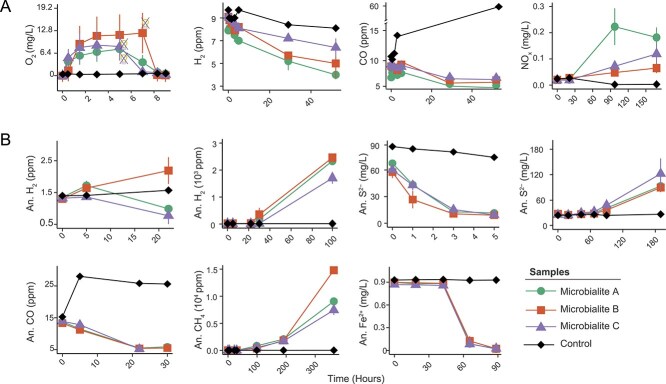
Biogeochemical assays illustrating the metabolic activities of microbialite communities under aerobic (**A**) and anaerobic (**B**) conditions in incubations. Oxygen dynamics were assessed using chemical imaging on independent microbialite samples incubated in 4 L glass aquaria, with data presented as the mean ± standard deviation for representative time points of defined photosynthetic regions of interest. Oxygen dynamics for each region of interest throughout the entire experiment are presented in supplementary Fig. 3. Trace gases, sulfide and ferrous ion measurements were taken in 120 ml sealed serum vial containing 10 g of microbialite slurry and 50 ml of 0.22 μm-filtered lake water. Trace gas incubations were supplemented with 10 ppm H_2_, CH_4_, and CO in the headspace. S^2−^ and Fe^2+^ incubations were supplied with either 100 μM Na₂S·9H₂O (only for consumption) or 6 mM FeCl₂. All anaerobic incubations except for S^2−^ production were supplemented with 1.5 mM NO_3_^−^ as an electron acceptor. Nitrification (NO_x_ = NO_2_^−^ + NO_3_^−^) measurements were taken in uncapped 250 ml Schott bottles containing ~10 g of microbialite slurry, 100 ml of 0.22 μm-filtered lake water and 100 μM NH_4_^+^. In the oxygen plot, crossed-off light bulbs indicate the time when light was switched off, mimicking the onset of darkness. All incubation experiments were performed in triplicate, with results expressed as the mean ± standard deviation across three replicates.

To validate our biogeochemical predictions, we performed ex situ incubation experiments. The potential for oxygenic photosynthesis was assessed using chemical imaging that simultaneously visualises and quantifies O_2_ concentrations in natural samples [88] (detailed in “Chemical Imaging”; Materials and Methods). To simulate the diel cycle, microbialite samples were exposed to an environmentally relevant light intensity of 450 μmol m^−2^ s^−1^ for ~7 hours, followed by a minimum of 6 hours of darkness. Algal and cyanobacterial populations, abundant in the microbialite communities, produced substantial O_2_ in the surficial microbialite layers creating oxic microenvironments (Fig. 3A; Supplementary Fig. 3). However, gross O_2_ production varied markedly both within individual samples (e.g. Sample 2 ranged from 29 to 182 μmol O_2_ L^−1^ min^−1^) and among samples (Fig. 3A; Supplementary Fig.3). Upon transition to darkness, O_2_ was rapidly depleted, primarily through aerobic respiration and diffusion into the overlying water column (Fig. 3A). We previously observed a similar process in the coral skeleton [88], where O_2_ produced during the day by endolithic algae diffuses through the porous CaCO_3_ skeleton creating transient oxic microenvironments, but upon the onset of darkness is quickly consumed through microbial respiration. Similarly, in microbialites, rapid O_2_ consumption likely contributes to shaping niches for the widespread facultative and obligate anaerobes at the millimetre-scale (Supplementary Data 2; Supplementary Fig. 3). In addition to supporting aerobic organotrophic respiration, the O_2_ generated via oxygenic photosynthesis serves as an electron acceptor for several lithotrophic processes, including hydrogenotrophy, carboxydotrophy, and nitrification. Correspondingly, all samples demonstrated aerobic oxidation of H_2_, CO, and NH_4_^+^, albeit at varying rates (Fig. 3A).

We observed consumption of H_2_, CO, Fe^2+^, and S^2−^ in nearly all anaerobic incubations (Fig. 3B), demonstrating the collective metabolic versatility of the microbialite community. The consumption of these substrates is likely coupled to anaerobic respiratory chains such as sulfate reduction and denitrification. Diverse electron acceptors were present in the microbialite samples, including high concentrations of SO_4_^2−^ (67 850 ± 35 100 μg/L) and moderate concentrations of NO_3_^−^ (200 ± 1.5 μg/L; Supplementary Data 1). Although these measurements do not resolve whether individual microbial taxa are specialised for specific substrates or capable of metabolising multiple ones, they underscore the high activity and diversity of metabolic pathways at the community level. This metabolic versatility aligns with genomic predictions made by previous studies [26, 52, 89] and reflects the capacity of West Basin Lake microbialite communities to exploit alternative electron donors and acceptors to sustain their nutrient web (Fig. 1). The extensive metabolic capacity enables the community to optimise energy conservation across the dynamic physicochemical gradients of the microbialite environment.

Our genomic predictions further indicate that microbialites appear to host microbial populations with high capacity for reductive metabolisms (Fig. 1). Accordingly, incubation experiments under prolonged anoxia confirmed the production of large amounts of reduced compounds (Fig. 3B). Despite methanogens being low in abundance, their elevated activity enables them to substantially contribute to CH_4_ production (29.8 ± 10.1 ppm h^−1^ g_wet_^−1^; Fig. 3B), a characteristic previously documented in other environments [90]. Similarly, sulfate reducers were scarce (*asrA, dsrA:* 2.4 ± 2.3%) but released abundant S^2−^ (0.6 ± 0.1 mg/L h^−1^ g_wet_^−1^; Fig. b). In contrast, the high abundance of hydrogenogenic fermenters ([FeFe]-hydrogenase: 34.4 ± 4.4%; group 3 [NiFe]-hydrogenase: 48.8 ± 7.1%) and nitrogen fixers (*nifH:* 27.5 + 7.1%) was mirrored in the high rates of H_2_ production (22.7 ± 3.8 ppm h^−1^ g_wet_^−1^; Fig. 3B). These reduced compounds, diffusing through the microbialite structure, fuel a diverse array of aerobic and anaerobic metabolic pathways, thereby supporting community-level energy conservation and carbon fixation.

### Chemosynthesis and photosynthesis contribute significantly to carbon fixation

**Figure 4:**
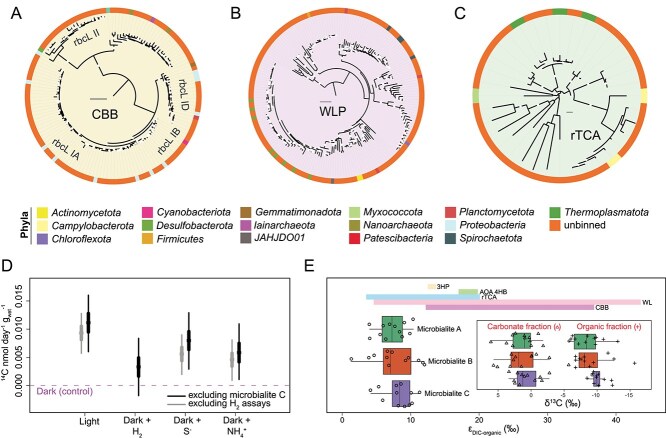
Dominant carbon fixation pathways and activities in microbialite communities. Maximum-likelihood phylogenetic trees of 140 RbcL (**A**), 184 AcsB (**B**), and 36 AclB (**C**) amino acid sequences obtained from the three microbialite samples, constructed using 1000 ultrafast bootstrap replicates. The LG + R5 (**A**), LG + F + I + R6 (**B**), and LG + I + G4 (**C**) substitution models were applied. Sequences derived from binned contigs are classified at the phylum level. Phylogenetic trees scale bars are 0.1. Bootstrap support values ≥90 are denoted by white circles (**A–C**). ^14^CO_2_ incorporation by microbialite incubations supplemented with different energy sources (light [40 μmol m^−2^ s^−1^], H_2_ [100 ppm], S^2−^ [0.8 mM], and NH_4_^+^ [1 mM]) (**D**). Credible intervals of effect (electron donors) relative to dark condition denoting 2.5th and 97.5th (thin bar) and 25th and 75th (thick bar) percentiles of posterior probability distribution (**D**). The rates do not represent gross carbon fixation rates due to the presence of unlabelled native inorganic carbon (average ~5.57 ± 1.4%; Supplementary Data 1) and internally recycled CO_2_ within samples (**D**). Please refer to materials and methods section “^14^C incorporation analysis” for detailed information on the hierarchical Bayesian model used to analyse these data and the reasoning behind the exclusion of microbialite C and H_2_ assays (**D**). Boxplot showing the carbon isotope fractionation (ε_DIC-organic_ in ‰, Vienna pee Dee belemnite standard) between dissolved inorganic carbon (DIC) and organic carbon of three independent microbialites (12 replicates each) (**E**). Note that the reported fractionation factor of 2.7‰ for aragonite [57] was used to estimate δ^13^C of DIC from δ^13^C of carbonate fraction, though the primary mineral of sampled microbialites is more likely to be hydromagnesite (**E**). Enclosed boxplot shows the natural abundance of carbon isotope compositions (δ^13^C) of the carbonate and organic fractions of the microbialites (**E**). Coloured bars depict the range of literature ε_DIC-organic_ values of cellular biomass produced from 3-hydroxypropionate cycle [58, 59] (3HP), 4-hydroxybutyrate cycle of ammonia oxidising archaea [60–62] (AOA 4HB), reductive tricarboxylic acid cycle [59, 63, 64] (rTCA), wood–Ljungdahl pathway [59] (WL), and Calvin-Benson-Bassham cycle [53, 58, 59] (CBB) (**E**).

Recent genomic studies suggest chemosynthetic pathways supplement primary production in hypersaline microbial mats [10, 26, 28]. Despite there is long-standing evidence for photosynthesis in microbial mats [91–93], experimental evidence for chemolithoautotrophy is fragmentary. To gain a comprehensive understanding of the diversity of microbialite autotrophic populations, we performed phylogenetic analyses of carbon fixation protein sequences (Fig. 4A-C; Supplementary Fig. 4). These phylogenetic inferences were further supported by activity measurements using ^14^C incorporation assays conducted with a range of supplemental electron donors, and ^13^C/^12^C organic and inorganic fractionation profiling (Fig. 4D-E).

The phylogenies of amino acid sequences of the acetyl-CoA synthase (AcsB), ribulose 1,5-bisphosphate (RbcL), and ATP-citrate lyase (AclB; Fig. 4A-C) revealed a diversity of 140 RbcL, 184 AcsB, and 36 AclB amino acid sequences affiliated with at least 16 phyla across the CBB, WLP, and rTCA, respectively (Fig. 4A-C). These sequences encompassed several previously uncharacterised autotrophic clades, including two *Nanoarchaeota* MAGs encoding the CBB cycle*,* one *JAHJDO01* MAG encoding the WLP, and one *Polyangia* MAG encoding the rTCA cycle (Fig. 4A-C). Across the 140 RuBisCO sequences, we recovered four subtypes (Fig. 4A). RuBisCO subtypes IA-D are known for their intermediate to high specificity for CO_2_ and are generally found across both oxic and anoxic habitats, whereas RuBisCO subtype II, which has lower CO_2_ specificity, is more commonly associated with anaerobic or microaerophilic environments [94]. Consistent with the prevalent anoxic niches of most microbialites, we recovered 184 AcsB amino acid sequences for the WLP (Fig. 4B). However, a large proportion of these sequences most likely were not encoded by autotrophs but rather by members of the phyla *Actinomycetota*, *Desulfobacterota*, and *Chloroflexota* that could use the reverse WLP for acetate oxidation coupled with dissimilatory sulfate reduction [23]. Conversely, sequences associated with *Firmicutes*, *Planctomycetota*, and *Spirochaetota* likely represent carbon-fixing acetogens, as demonstrated by prior studies characterising these phyla [95, 96]. The rTCA cycle may also significantly contribute to carbon fixation within the community, with the majority of AclB sequences associated with the phyla *Myxococcota* and *Campylobacterota* (Fig. 4C).

To validate the potential for carbon fixation inferred through genomic and phylogenetic analysis, we quantified relative chemosynthetic and photosynthetic carbon fixation rates using radiolabeled carbon dioxide (^14^CO_2_). To capture both aerobic and anaerobic carbon fixation processes, we conducted 5-day experiments, during which the incubations transitioned to anaerobic conditions after ~24 h. This transition was inferred based on O_2_ measurements taken simultaneously on parallel incubations. ^14^CO_2_ incorporation was detected in all incubations, except for the H_2_-stimulated chemosynthesis experiment in microbialite C (Fig. 4D, Supplementary Fig. 5), which failed, likely as a result of human error while adding ^14^CO_2_ to the incubations. As anticipated, incubations exposed to light supported higher ^14^CO_2_ incorporation (12.2 ± 4.9 pmol day^−1^ g_wet_^−1^) compared to those incubated in the dark (2.8 ± 2.0 pmol day^−1^ g_wet_^−1^; Fig. 4D). Dark incubations likely captured the combined activity of anaplerotic processes and baseline chemosynthetic carbon fixation. The addition of electron donors significantly boosted chemosynthetic carbon fixation rates (Fig. 4D). In line with the high abundance of the *sqr* gene (Fig. 1C), S^2—^supplemented incubations exhibited the highest chemosynthetic ^14^CO_2_ incorporation rates (8.4 ± 5.2 pmol day^−1^ g_wet_^−1^), equivalent to ~69% of ^14^CO_2_ of incorporated through photosynthesis. Similarly, NH_4_^+^ and H_2_ supplementation enhanced chemosynthetic carbon fixation (7.4 ± 4.9 and 6.2 ± 4.3 pmol day^−1^ g_wet_^−1^, respectively; Fig. 4D).

Finally we probed the origin of West Basin Lake microbialite organic matter by measuring the carbon isotopic composition (δ^13^C) of organic carbon and carbonate fractions. Carbon isotopic compositions of the carbonates (δ^13^C_carb_) were around 1.1‰ VPDB (Fig. 4E), consistent with a recent geochemical survey of microbialites [97]. Isotopic compositions of the organic carbon (δ^13^C_org_) were depleted in ^13^C relative to the carbonates, with values ranging from −5.7 to −15.7‰ (−9.3 ± 2.2‰; Fig. 4E); substantially smaller fractionations than is generally expected for organic matter from cyanobacterial or algal origin (20 to 30‰ depleted in ^13^C relative to DIC) [98–100]. One possible explanation is that a substantial portion of the bulk biomass is derived from autotrophic processes that are characterised by relatively small carbon isotope fractionations. In the microbialites of West Basin Lake, the degree of fractionation between organic and inorganic carbon (ε_DIC-organic_) would reflect contributions from the rTCA cycle and WLP (Fig. 4E). Extensive remineralisation of organic carbon, e.g. through respiration or fermentation that produces CO_2_ slightly isotopically depleted relative to the carbon of the food source [101], and could lead to δ^13^C_org_ enrichment of the residual organics. However, in this scenario the carbonate fraction would be expected to be ^13^C-depleted if precipitated from ^13^C-depleted DIC. Meanwhile, a relatively ^13^C-enriched organic fraction could also result from high utilisation of DIC, and a corresponding enrichment of the residual DIC pool; however, if carbonate production is contemporaneous, this enrichment would be expected to result in ^13^C-enriched carbonate. Therefore, although natural abundance carbon isotope values alone cannot definitively distinguish among carbon fixation pathways, the most parsimonious interpretation is that the rTCA cycle and the WLP make significant contributions to primary production. This is in line with the observed high abundance of genes and taxa that host these pathways among the West Basin Lake microbialite communities (Fig. 1) and the evidence from ^14^C fixation assay (Fig. 4D). We conclude that inorganic chemical sources and gas substrates are critical for the primary productivity of these ecosystems, highlighting the significant role of chemosynthetic carbon fixation pathways in supporting microbial mat communities.

## Conclusions

Microbialite communities in the hypersaline West Basin Lake exhibit a metabolic diversity comparable to that observed in other microbialite ecosystems worldwide. This diversity likely arises from the presence of organisms with complementary traits, driven by resource facilitation among community members [102, 103]. In microbialite communities, metabolic synergies occur between organisms inhabiting contrasting physicochemical niches [26, 104], which vary on a diel cycle. The resultant mosaic of ecological niches align with the physiological requirements of diverse microbial taxa. Consistent with these observations, our analysis simultaneously reveals millimetre-scale overlap of functional guilds throughout the microbialite structure and cycling of key metabolites such as iron, nitrogen, and sulfur compounds across their major redox states through intricate molecular handoffs. These processes minimise energy loss and enhance ecosystem productivity [105]. In a broader context, the exceptional efficiency of elemental cycling and carbon use efficiency within microbialite communities, combined with the duality of light- and chemically-driven metabolic pathways, suggests that these ecosystems have likely served as hotspots of metabolic innovation throughout Earth’s history.

## Supplementary Material

Supp_Data_1_-_physicochemistry_wraf117

Supp_Data_2_-_16S_rRNA_gene_wraf117

Supp_Data_3_-_MAGs

Supp_Data_4_-_pathways_completeness_wraf117

Supp_Data_5_-_short_reads_analysis_wraf117

Supp_Data_6_-_fermentation_genes

Supp_Data_7_-_mcrA_stats

supplementary_figures_1-5_wraf117

Legends_wraf117

## Data Availability

All data supporting the findings of the present study are available. All sequences generated from this work were deposited to the NCBI Sequence Read Archive. BioProject accession numbers for metagenomes, 16S rRNA gene amplicons, and metagenome-assembled genomes are PRJNA1194634, PRJNA1194668, and PRJNA1196970, respectively.
