# Supplementary material for: Chemosynthesis enhances net primary production and nutrient cycling in a hypersaline microbial mat
Source: ISME J. 2025 Jun 9;19(1):wraf117. doi: 10.1093/ismejo/wraf117 (PMC12218205; doi:10.1093/ismejo/wraf117)
Supplement: supplementary_figures_1-5_wraf117 [file supplementary_figures_1-5_wraf117.pdf]

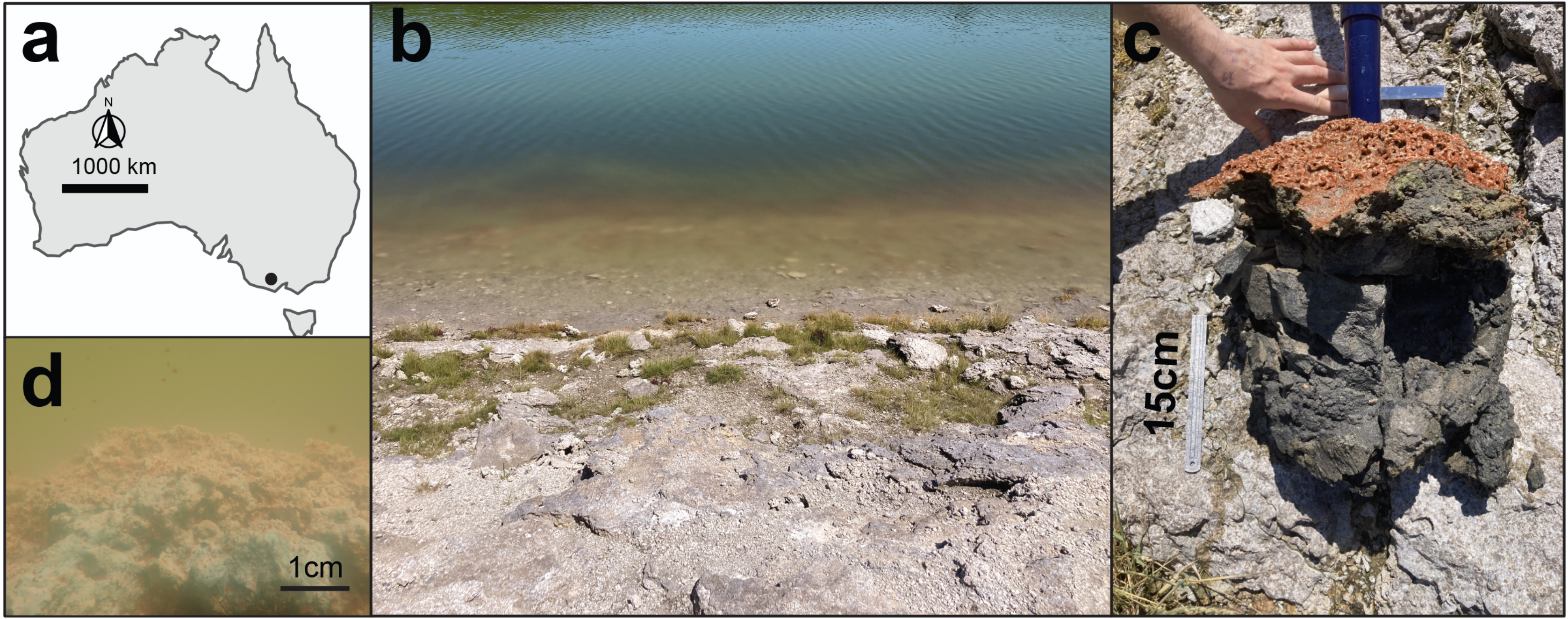
a
N
1000 km
b
c
15cm
d
1cm

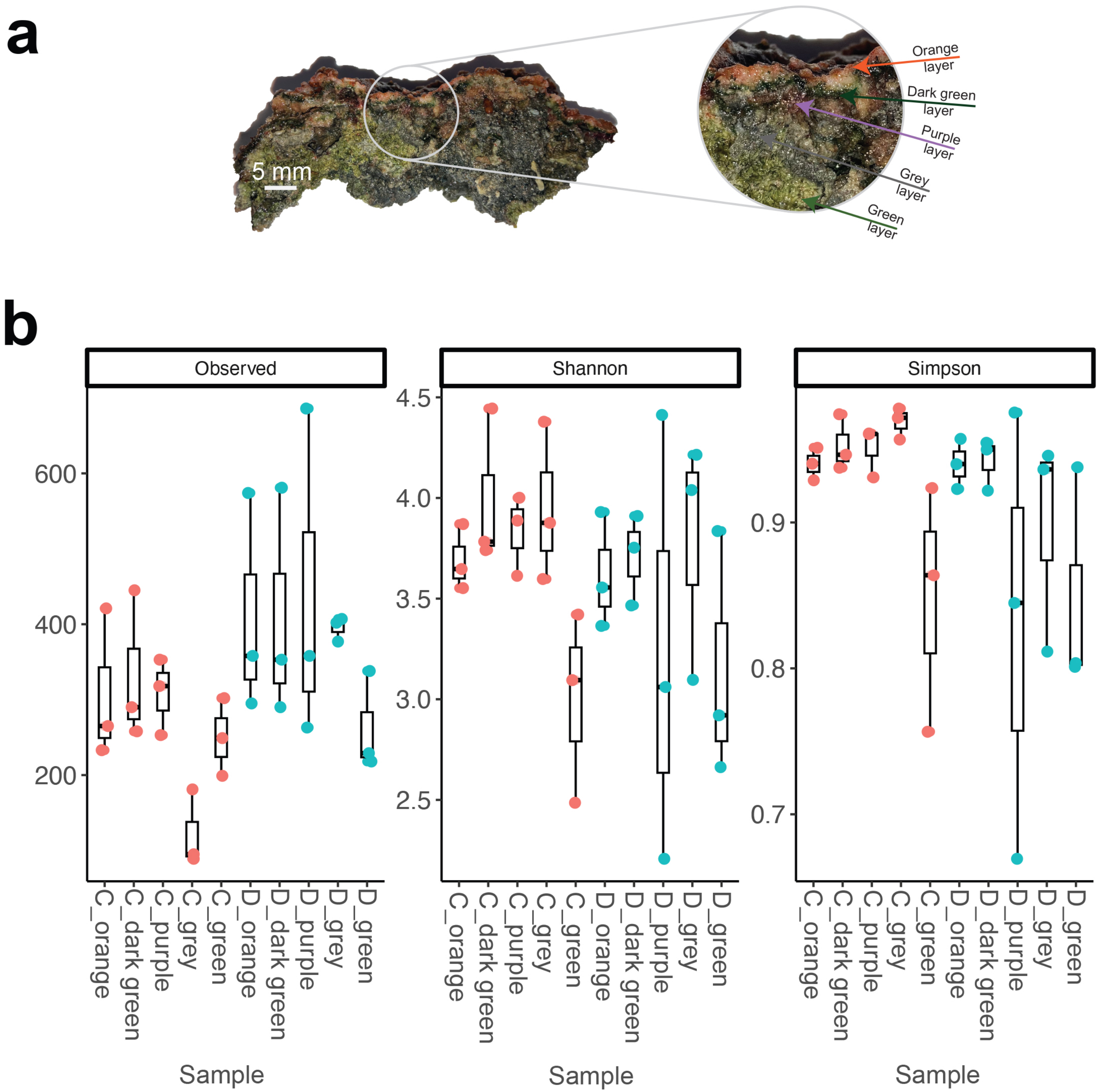

a
5 mm
Orange layer
Dark green layer
Purple layer
Grey layer
Green layer
b
Observed
Shannon
Simpson
600
400
200
4.5
4.0
3.5
3.0
2.5
0.9
0.8
0.7
C_orange
C_dark green
C_purple
C_grey
C_green
D_orange
D_dark green
D_purple
D_grey
D_green
Sample

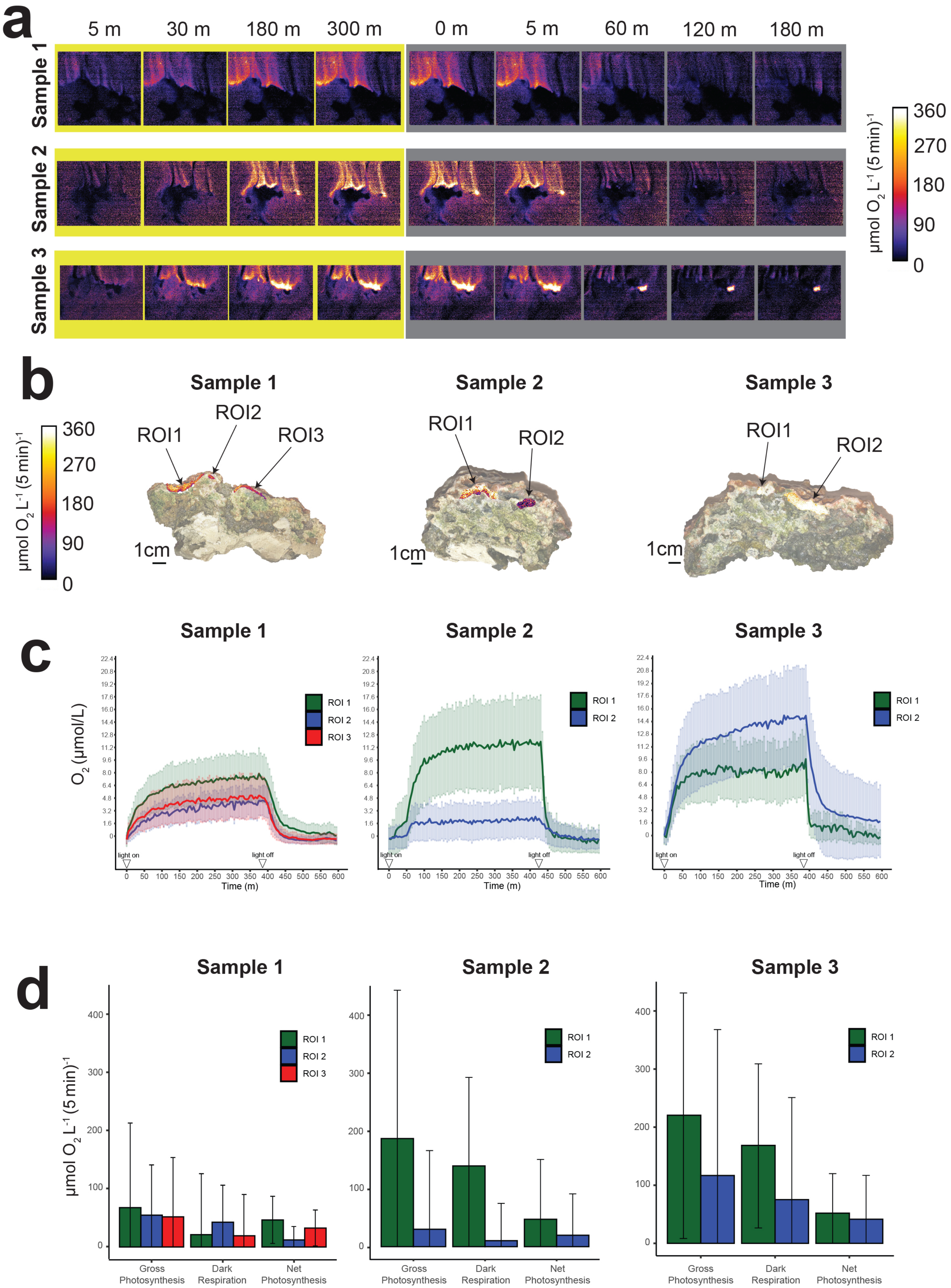
a
5 m
30 m
180 m
300 m
0 m
5 m
60 m
120 m
180 m
Sample 1
Sample 2
Sample 3
µmol O2 L-1 (5 min)-1
360
270
180
90
0
b
Sample 1
Sample 2
Sample 3
ROI1
ROI2
ROI3
1cm
c
O2 (µmol/L)
ROI 1
ROI 2
ROI 3
light on
light off
Time (m)
d
µmol O2 L-1 (5 min)-1
Gross Photosynthesis
Dark Respiration
Net Photosynthesis

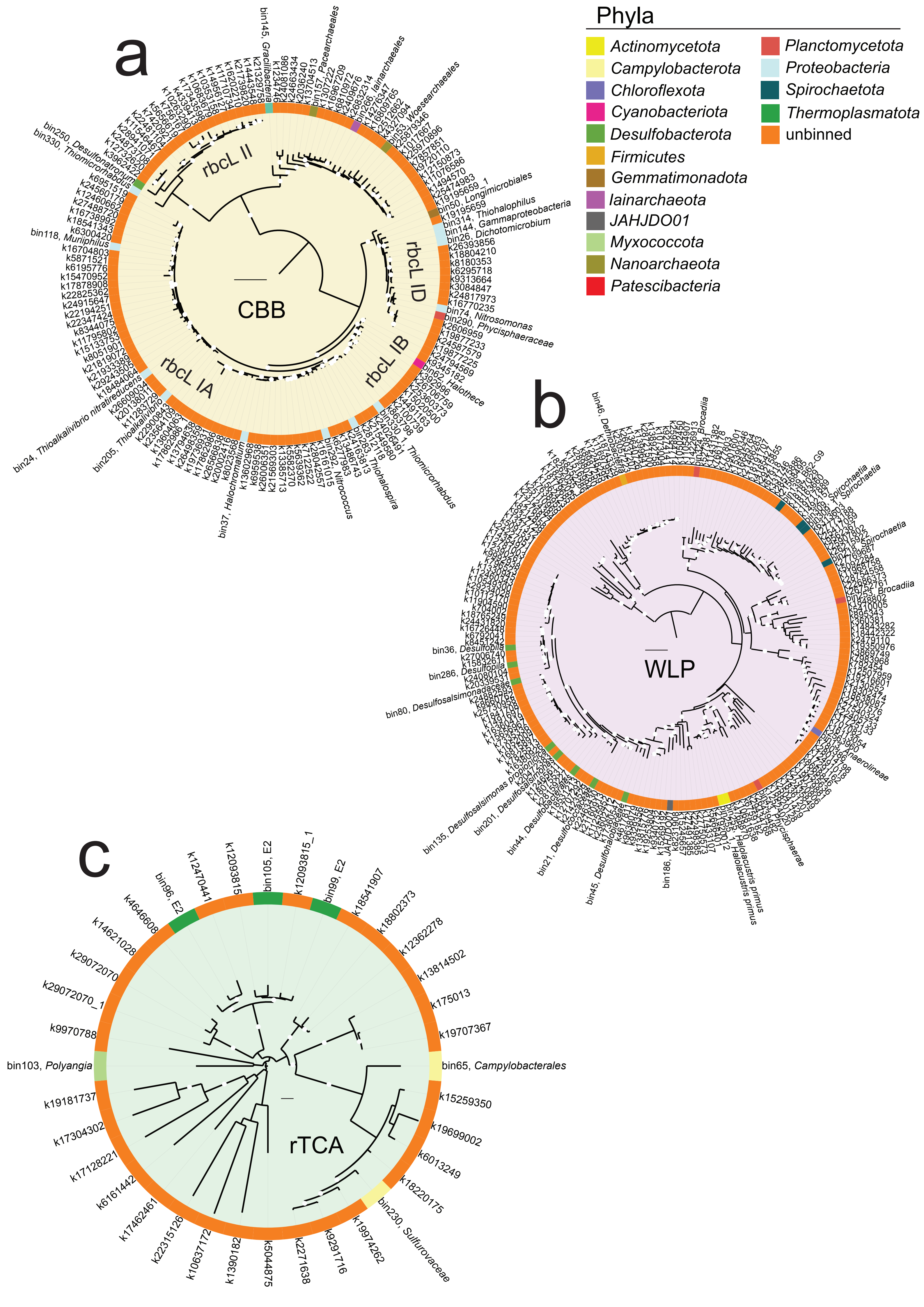

Phyla
Actinomycetota
Campylobacterota
Chloroflexota
Cyanobacteriota
Desulfobacterota
Firmicutes
Gemmatimonadota
Iainarchaeota
JAHJDO01
Myxococcota
Nanoarchaeota
Patescibacteria
Planctomycetota
Proteobacteria
Spirochaetota
Thermoplasmatota
unbinned
a
CBB
rbcL II
rbcL ID
rbcL IB
rbcL IA
bin145, Gracilibacteria
bin157, Pacearchaeales
bin266, Iainarchaeales
bin53, Woesearchaeales
bin50, Longimicrobiales
bin314, Thiohalophilus
bin144, Gammaproteobacteria
bin26, Dichotomicrobium
bin74, Nitrosomonas
bin290, Phycisphaeraceae
bin62, Halothece
Thiomicrorhabdus
bin283, Thiohalospira
bin292, Nitrococcus
bin37, Halochromatium
bin205, Thioalkalivibrio
bin24, Thioalkalivibrio nitratireducens
bin118, Muriiphilus
bin330, Thiomicrorhabdus
bin250, Desulfonatronum
b
WLP
bin46, Dethiobacter
bin324, Brocadiia
Spirochaetia
Spirochaetia
Spirochaetia
bin253, Brocadiia
Anaerolineae
Phycisphaerae
Halolacustris primus
Halolacustris primus
bin186, JAHJDO01
bin45, Desulfohalobiaceae
bin21, Desulfococcaceae
bin44, Desulfobacterales
bin201, Desulfosalsimonas
bin135, Desulfosalsimonas propionicica
bin80, Desulfosalsimonadaceae
bin286, Desulfopila
bin36, Desulfopila
c
rTCA
bin105, E2
k12093815_1
bin99, E2
k18541907
k18802373
k12362278
k13814502
k175013
k19707367
bin65, Campylobacterales
k15259350
k19699002
k6013249
k18220175
bin230, Sulfurovaceae
k19974262
k9291716
k2271638
k5044875
k1390182
k10637172
k22315126
k17462461
k6161442
k17128221
k17304302
k19181737
bin103, Polyangia
k9970788
k29072070_1
k29072070
k14621028
k4646608
bin96, E2
k12470441
k12093815

a

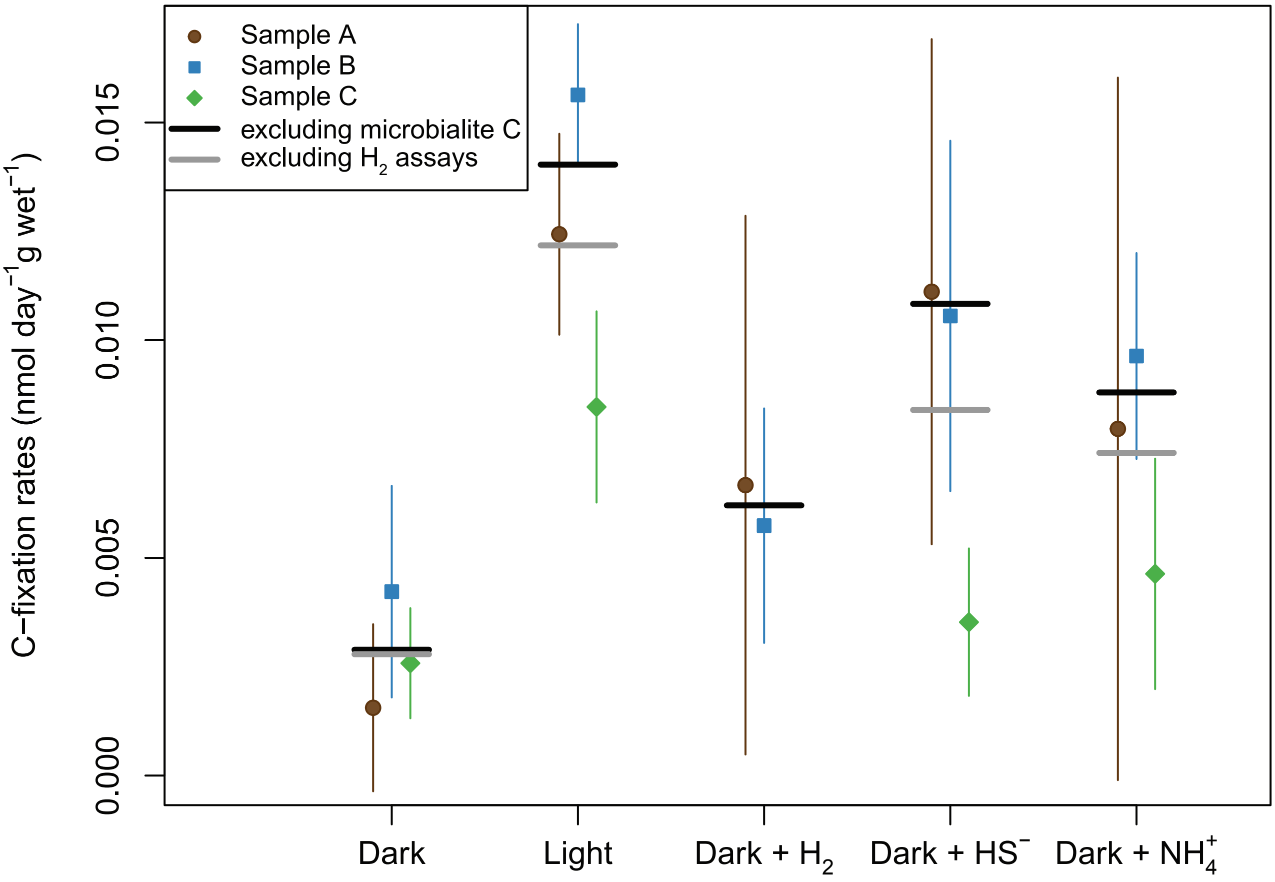

Sample A
Sample B
Sample C
excluding microbialite C
excluding H_2 assays
C−fixation rates (nmol day^{-1}g wet^{-1})
0.000
0.005
0.010
0.015
Dark
Light
Dark + H_2
Dark + HS^-
Dark + NH_4^+

Supplementary Figures

Supp. Figure 1 | (**a**) Location of West Basin Lake on Australian map. (**b**) Picture showing portion of the microbialite reef (in red) at West Basin Lake. (**c**) Example of a freshly collected microbialite. (**d**) Close-up image of a microbialite underwater.

Supp. Figure 2 | (**a**) Cross-section of a microbialite with a close-up view highlighting the five sub-samples collected across its structure. (**b**) Alpha diversity metrics, including Observed ASVs, Shannon and Simpson, comparing microbial communities from the layers (x-axis) of two microbialite samples, with sample C in red and sample D in light blue.

Supp. Figure 3 | (**a**) Chemical imaging analysis showing O_2 dynamics during surficial homogeneous light exposure (yellow background) and after the onset of darkness (grey background) of three microbialite cross-section samples. (**b**) Photosynthetic region of interest (ROI) data obtained via chemical imaging, overlaid onto microbialite samples cross sections. (**a**-**b**) The ROI colour scale corresponds to the scale bar indicating oxygen concentration expressed in µmol O_2 L^{-1} (5 min)$^{-1}$. (**c**) Oxygen dynamics for each ROI throughout the entire experiment presented as the mean ± standard deviation of each data point. (**d**) Bar graphs illustrating rates of gross photosynthesis, dark respiration and net photosynthesis within the photosynthetic ROI of each microbialite sample. Data are presented as mean ± standard deviation across photosynthetic ROI. Detailed methods describing gross photosynthesis, dark respiration, and net photosynthesis calculations are reported in the Materials and Methods section Chemical Imaging.

Supp. Figure 4 | Dominant carbon fixation pathways and activities in microbialite communities. Maximum-likelihood phylogenetic trees were constructed for 140 RbcL (**a**), 184 AcsB (**b**), and 36 AclB (**c**) amino acid sequences obtained from three microbialite samples, using 1,000 ultrafast bootstrap replicates. The substitution models applied were LG+R5 for RbcL (**a**), LG+F+I+R6 for AcsB (**b**), and LG+I+G4 for AclB (**c**). (**a–c**) Sequences derived from binned contigs are classified at the phylum level, whereas those from unbinned contigs are displayed in orange. Scale bars represent 0.1 substitutions per site. Bootstrap support values ≥ 90 are indicated by white circles.

Supp. Figure 5 | Plot illustrating ^{14}C incorporation across nine technical replicates of the three microbialite samples exposed to five experimental conditions.
