# Supplementary material for: Chemosynthesis enhances net primary production and nutrient cycling in a hypersaline microbial mat
Source: ISME J. 2025 Jun 9;19(1):wraf117. doi: 10.1093/ismejo/wraf117 (PMC12218205; doi:10.1093/ismejo/wraf117)
Supplement: Legends_wraf117 [file legends_wraf117.docx]

# Legends

## Supplementary Figures

**Supp. Figure 1 |** (**a**) Location of West Basin Lake on Australian map. (**b**) Picture showing portion of the microbialite reef (in red) at West Basin Lake. (**c**) Example of a freshly collected microbialite. (**d**) Close-up image of a microbialite underwater.

**Supp. Figure 2 |** (**a**) Cross-section of a microbialite with a close-up view highlighting the five sub-samples collected across its structure. (**b**) Alpha diversity metrics, including Observed ASVs, Shannon and Simpson, comparing microbial communities from the layers (x-axis) of two microbialite samples, with sample C in red and sample D in light blue.

**Supp. Figure 3 |** (**a**) Chemical imaging analysis showing O_2_ dynamics during surficial homogeneous light exposure (yellow background) and after the onset of darkness (grey background) of three microbialite cross-section samples. (**b**) Photosynthetic region of interest (ROI) data obtained via chemical imaging, overlaid onto microbialite samples cross sections. (**a**-**b**) The ROI colour scale corresponds to the scale bar indicating oxygen concentration expressed in μmol O_2_ L^-1^ (5 min)^-1^. (**c**) Oxygen dynamics for each ROI throughout the entire experiment presented as the mean ± standard deviation of each data point. (**d**) Bar graphs illustrating rates of gross photosynthesis, dark respiration and net photosynthesis within the photosynthetic ROI of each microbialite sample. Data are presented as mean ± standard deviation across photosynthetic ROI. Detailed methods describing gross photosynthesis, dark respiration, and net photosynthesis calculations are reported in the Materials and Methods section Chemical Imaging.

**Supp. Figure 4 |** Dominant carbon fixation pathways and activities in microbialite communities. Maximum-likelihood phylogenetic trees were constructed for 140 RbcL (**a**), 184 AcsB (**b**), and 36 AclB (**c**) amino acid sequences obtained from three microbialite samples, using 1,000 ultrafast bootstrap replicates. The substitution models applied were LG+R5 for RbcL (**a**), LG+F+I+R6 for AcsB (**b**), and LG+I+G4 for AclB (**c**). (**a–c**) Sequences derived from binned contigs are classified at the phylum level, whereas those from unbinned contigs are displayed in orange. Scale bars represent 0.1 substitutions per site. Bootstrap support values ≥ 90 are indicated by white circles.

**Supp. Figure 5 |** Plot illustrating ^14^C incorporation across nine technical replicates of the three microbialite samples exposed to five experimental conditions.

## Supplementary Tables

**Supp. Data 1** **|** Table showing the relative abundance of bacterial and archaeal ASVs, as well as the ASV count of eukaryotes, based on the 16S rRNA gene dataset across the layers of two microbialite samples. Results of PERMUTEST and PERMANOVA analyses on beta diversity among microbialite layers are also included.

**Supp. Data 2** **|** Table highlighting the presence of 57 marker genes across the 331 MAGs generated in this study.

**Supp. Data 3** **|** Table showing lake and microbialites physicochemical parameters, namely C, N, Na, Mg, K, SO_4_^2-^, Cl^-^, NO_3_, and ^13^C.

**Supp. Data 4 |** Table showing pathways completeness across the metagenome-assembled genomes.

**Supp. Data 5** **|** Table presenting metagenomic short read data across three West Basin Lake microbialite samples and 17 publicly available microbialite samples from five global sites.

**Supp. Data 6 |** Table showing genes encoded in pathways involved in acetate, formate and lactate cycling recovered in the metagenome-assembled genomes.

**Supp. Data 7 |** Table showing *mcrA* gene statistics across the microbialite samples.
